# Risk of leakage with a new detachable multi-hard-port containment system for power morcellation during gynecologic laparoscopy: An in vitro study

**DOI:** 10.1186/s12893-023-02124-1

**Published:** 2023-07-31

**Authors:** Fang Zhao, Wenhui Wang, Bin Ling, Jing Liang

**Affiliations:** Department of Obstetrics and Gynecology, China-Japan Friendship Hospital, Beijing, 100029 China

**Keywords:** Laparoscopy, Power morcellation, Myomectomy, Tissue extraction

## Abstract

**Background:**

Laparoscopic surgery has been a milestone for minimally invasive surgeries. But safe removal of large uterine tissue is a challenge for minimally invasive procedures, and there still exists concern about the dissemination of benign or occult malignant uterine tissue during the use of the morcellator. Different tissue containment systems have been used in laparoscopic power morcellation. However, a risk of leakage still exists in clinical practice. In this study, we aimed to evaluate leakage and tissue dissemination associated with a new detachable multi-hard-port containment system for tissue removal during laparoscopic myomectomy morcellation.

**Methods:**

Beef tongue specimens were stained with methylene blue solution and morcellated in a plastic trainer box under laparoscopic guidance. The morcellation test in vitro conditions comprised two different containment systems to simulate laparoscopic power morcellation, specifically a polyurethane bag with two pipes (control group) and a detachable multi-hard-port containment system (experimental group). Insufflation pressure was set at 14 mmHg. Three methods are used to detect the leakage The procedure times were recorded. Thirty trials were performed using a multi-port approach and the two tissue containment systems.

**Results:**

The leakage rate was 0.03% (*n* = 30) for the experimental group and 26.6% (*n* = 30) for the control group (*p* < 0.005). Morcellation time was significantly shorter in the experimental group than in the control group (*p* < 0.001). Median bag introduction time was shorter in the experimental group than in the control group; however, removal time differences were not significant.

**Conclusions:**

This study quantified the low leakage rate during morcellation and the improved convenience of operation provided by a new tissue containment system.

## Background

In gynecology, Power morcellation is used to remove large uterine tissue from the abdominal cavity through small abdominal incisions. However, tissue disruption is unavoidable during morcellation

There is particular concern regarding the spread of morcellated tissue fragments in occult malignancy [1–4]. The US Food and Drug Administration (FDA) issued a safety communication in April 2014, discouraging the use of laparoscopic power morcellation during hysterectomy or myomectomy to treat uterine myomas [5]. In 2020, the FDA recommended performing laparoscopic power morcellation for myomectomy or hysterectomy with a tissue containment system compatible with the laparoscopic power morcellator [6]. Since then, various tissue containment systems have been developed, and studies related to their use have been conducted [7, 8]. However, different containment systems can result in leakage during multiple steps in clinical practice. We assessed the leakage and convenience associated with a new detachable multi-hard-port containment system for power morcellation.

## Methods

**Fig. 1:**
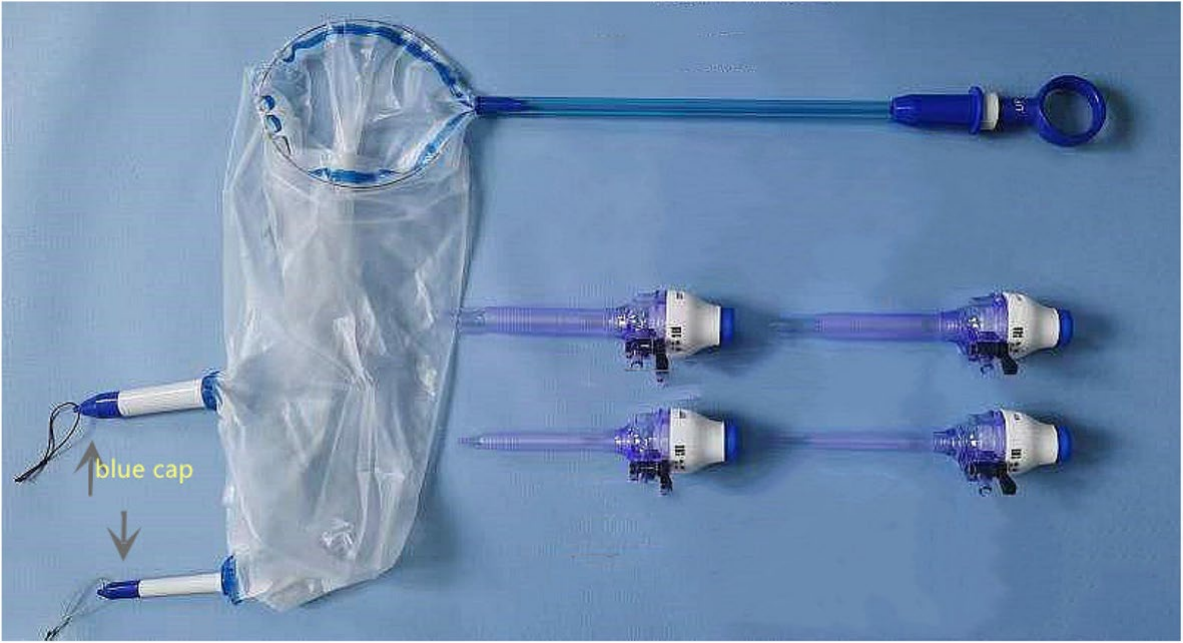
The system of the experimental group

**Fig. 2:**
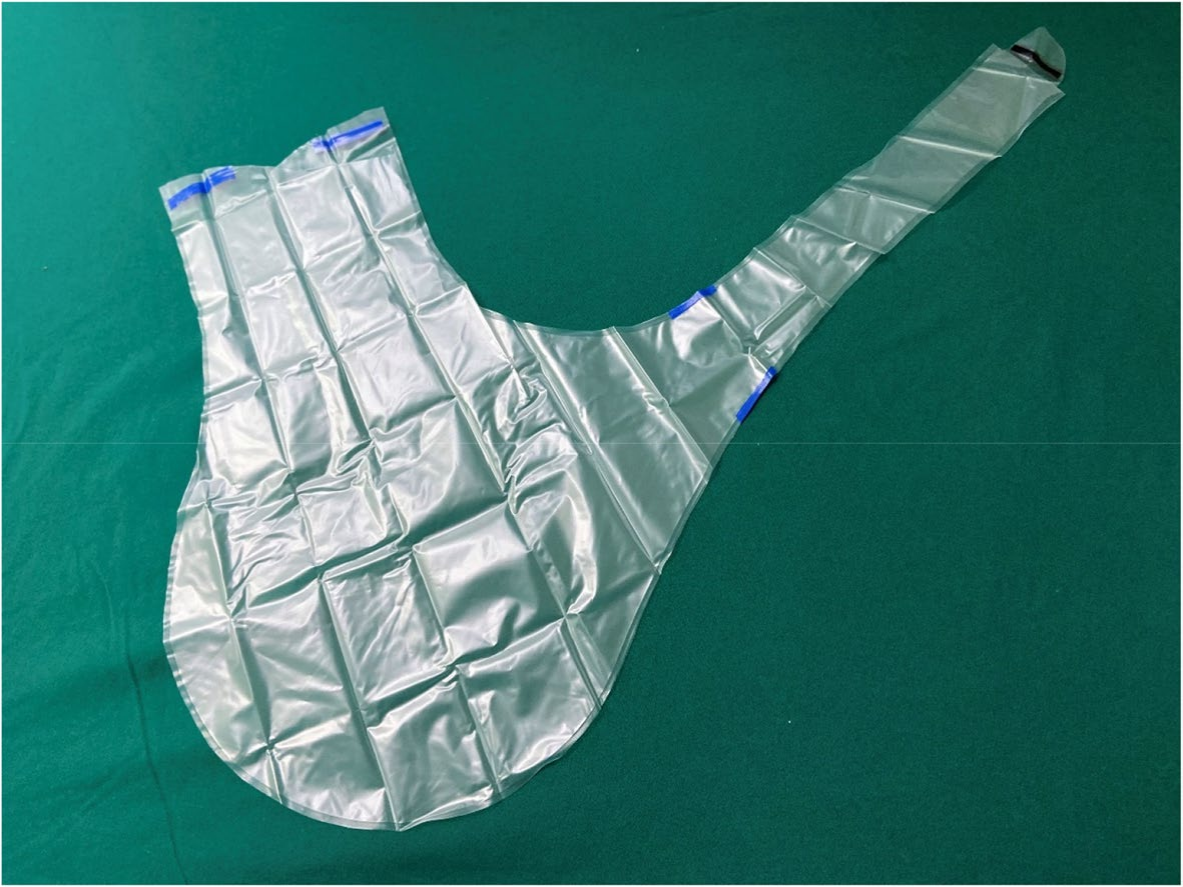
The system of the control group (Kangji Medical Holdings Limited, Grand Cayman, Cayman Islands, the United Kingdom)

This was a pilot study performed in an academic hospital laparoscopic skills laboratory using beef tongue specimens and an enclosed laparoscopic training box to simulate laparoscopic power morcellation during hysterectomy or myomectomy. The beef tongue specimens were purchased from Tianjin Jingdong Daye Trading Co. LTD. Training boxes were covered with a 2 cm-thick silica gel to simulate the abdominal wall. In agreement with other in vitro studies [9, 10], the beef tongue specimens were cut into 400 g sections. Pieces were dyed with 5 ml of methylene blue solution to aid leakage detection and morcellated in the tissue containment system. Two different tissue containment systems were evaluated. The new tissue containment system was the “experimental group” (Fig. 1). The second system in the control group (Kangji Medical Holdings Limited, Grand Cayman, Cayman Islands, the United Kingdom) consisted of a polyurethane bag, a 12 cm soft main pipe, and a 5 cm soft auxiliary pipe (Fig. 2). Details of the materials for the two groups of bags are listed in Table 1.

**Table 1:** Material and Leakage pressure for the two groups of bags

Bag	Material	Thickness, mm	Leakage pressure,psi
Experimental group	polyurethane	0.05 ± 0.001	> 25
Control group	polyurethane	0.06 ± 0.001	> 25

Before the trial, we randomly selected 10 systems in the experimental group and 10 systems in the control group for the tension test. First, inject 1000 ml of liquid into the bag to test for water leakage. Secondly, the bag body was inflated, and the pressure was adjusted to 20 mmHg ((the conventional pressure of laparoscopic artificial pneumoperitonea was 12-15 mmHg) to detect the expansion and integrity of the bag body. If the bag body cannot be fully expanded or leaks, it should be excluded due to the quality problems of the system itself. The results showed that all samples of the two systems passed the test and could enter the formal trial.

The new system in the experimental group had the following unique designs: 1. a detachable laparoscopic puncture sheath and multi-channel rigid pipes; 2. a sheath and ragid pipe that can be connected and disassembled repeatedly; 3. rigid pipes that are all equipped with a bullet-shaped sealing cap with a small ring on top that can be easily grasped by forceps; 4. the connecting part of the rigid pipe and the bag are equipped with an anti-slip device; 5. a bag opening which uses a metal ring connected with a movable double-layer stick to facilitate opening and tightening and has a blue logo for easy identification; 6. matching anti-slip and conformal fixing forceps for fixing the rigid pipe; 7. matching single-tooth grasping forceps for grasping rigid pipes. Thirty trials were performed using each tissue containment system with a multi-port approach. All physicians were gynecologic surgeons experienced at performing minimally invasive surgeries and had successfully completed over 20 in-bag-morcellation to ensure the similar experience with this techniques. Individual surgeons were randomly assigned to the experimental or control groups, and all trials were monitored by a senior surgeon.

**Fig. 3:**
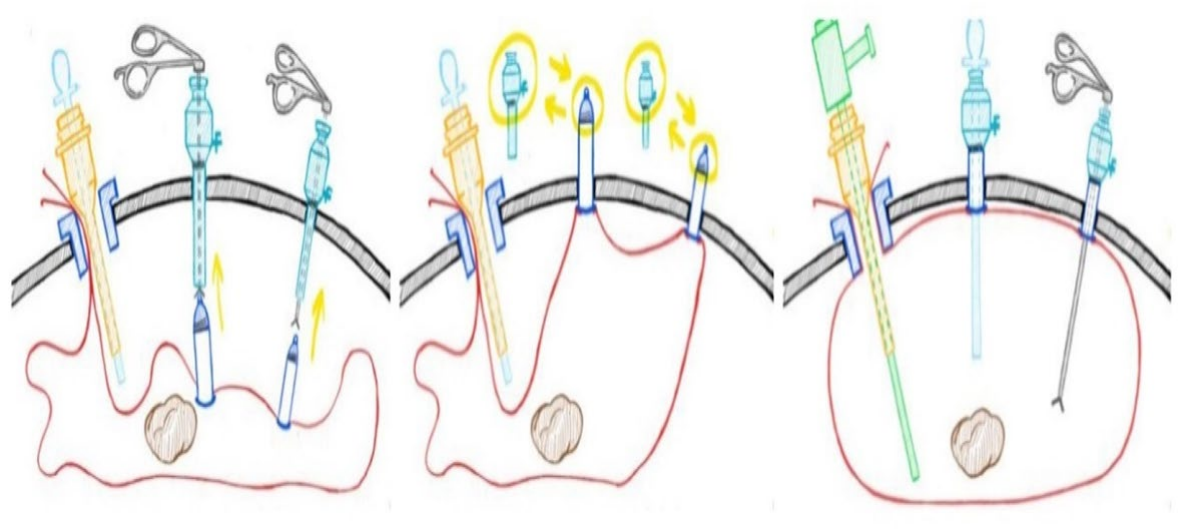
Morcellation under monitering in the experimental group

In the experimental group, the system was inserted into a training box via the right access with a 15 mm introduction sheath. In the box, the metal ring would open automatically, and the dyed specimen was placed into the bag. The opening of the bag was pulled out through the introduction sheath by approximately 2–3 cm. The top ring of the two rigid pipes was clamped with single-tooth grasping forceps and taken out through the umbilical access and left access. After the rigid pipe was fixed with anti-slip and conformal forceps, the sealing cap was removed, and it could be directly connected to the base of the laparoscopic sheath. After inflating the system through the laparoscopic sheath, an optical mirror was inserted through the umbilical access; then, assistant forceps and a morcellator were placed in the left and right access. With monitoring, the morcellation of the specimen was completed (Fig. 3), after which the optic mirror and forceps were removed, the bag was deflated, two rigid pipes were covered with sealing caps, and the pipes were inserted into the training box with reassembled trocars. Because the removed optic mirror and forceps may be stained, we now change the new optic mirror and forceps. Finally, the experimental system was removed via the right access under supervision.

In the control group, the system was inserted into the training box by clamping the soft bag via the right access, using forceps, through a 10-mm introduction sheath. In the box, the opening of the main pipe was opened with forceps, and the dyed specimen was placed into the bag. After the specimen was placed in the system, the edges of the pipe opening were clamped to close the bag with forceps, and the two soft pipes were removed from the right and umbilical access. The laparoscopic sheath was re-inserted into two soft pipes; then, an optical mirror and a morcellator were placed into the system through the laparoscopic sheath. The system was inflated through the laparoscopic sheath. After morcellation was completed, the optical mirror and morcellator were removed, the bag was deflated, and the pipe at the umbilical access was closed with two knots; then, it was re-inserted into the abdominal cavity. At this point, the control group also changed to a new optical mirror.Finally, the system was removed by pulling it via the right access.

**Fig. 4:**
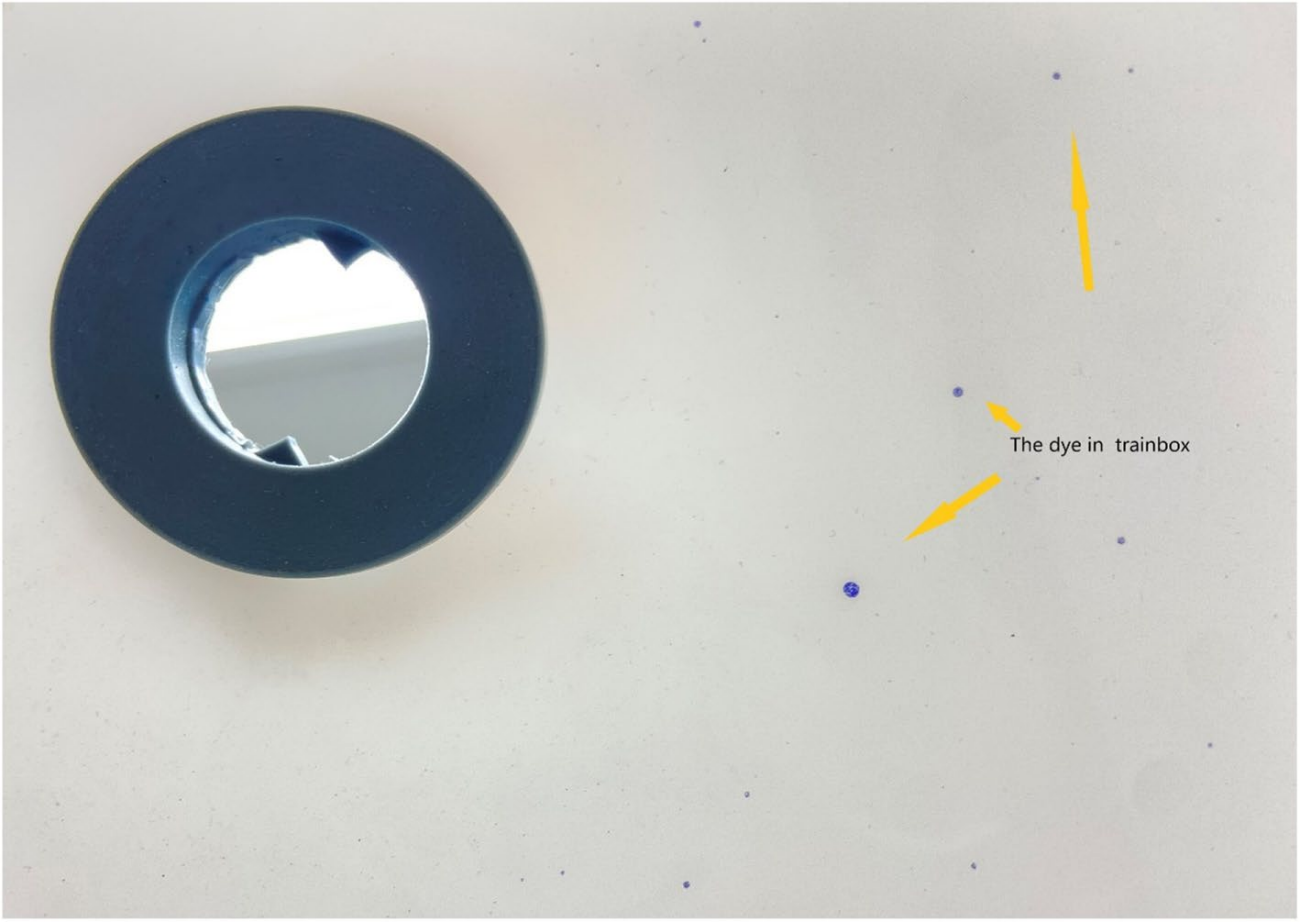
The dye in trainbox

**Fig. 5:**
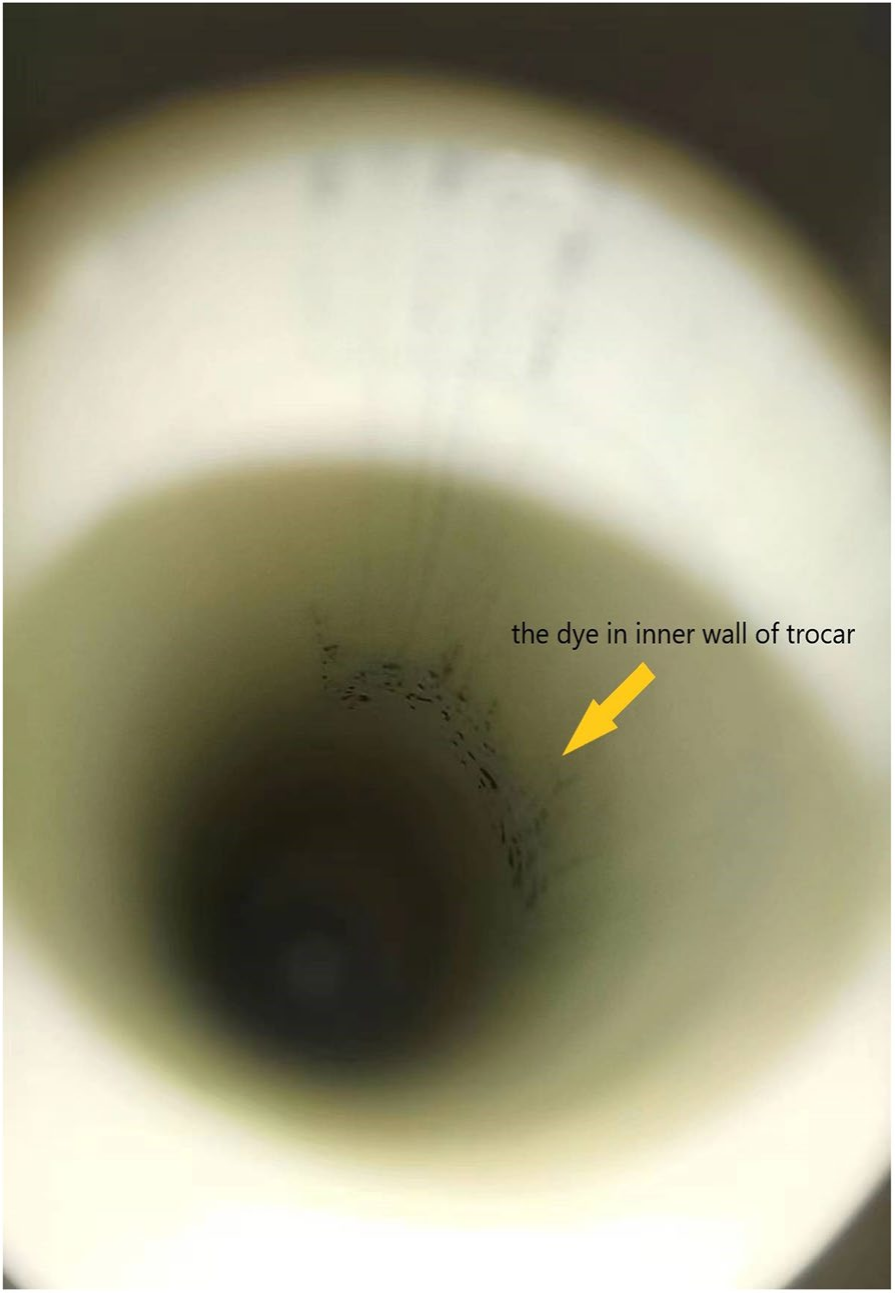
The dye in inner wall of the trocar

**Fig. 6:**
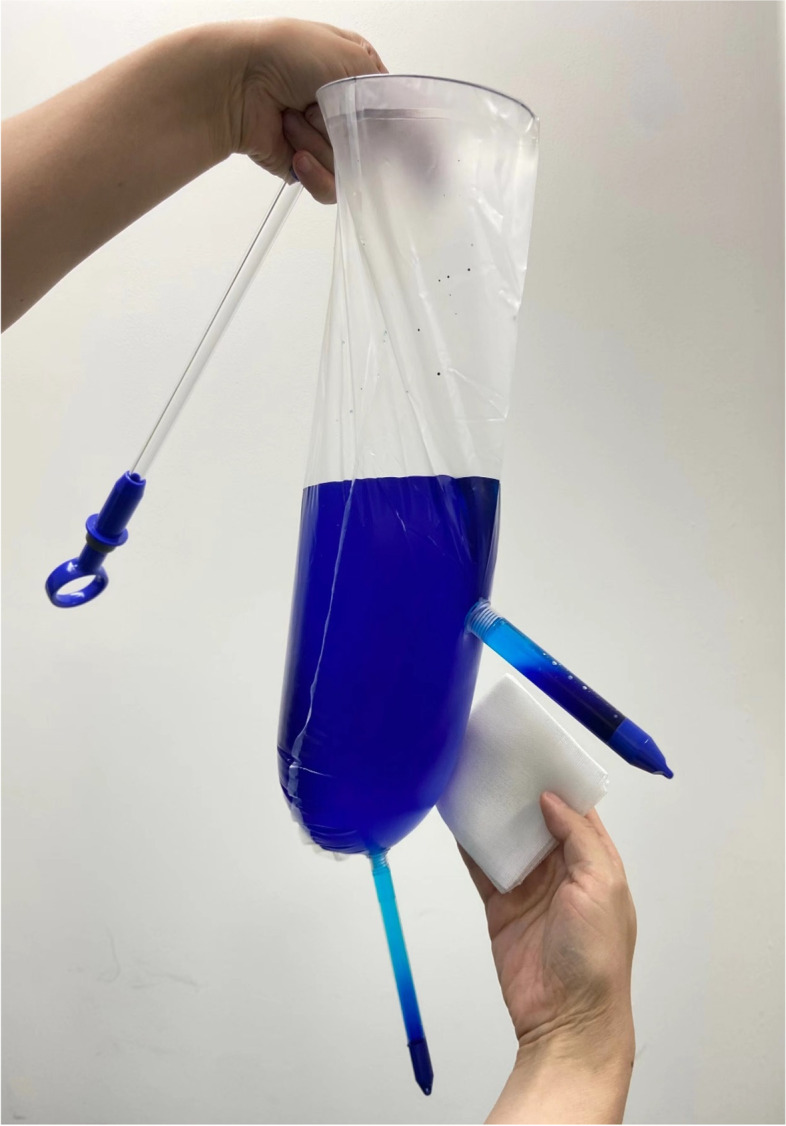
The detection of system demage

Three methods are used to detect the leakage The inner wall of the training box (Fig. 4) and trocar (Fig. 5) were examined for the presence of any dye. The systems were then filled with saline (1000 ml) to confirm the absence of damage (Fig. 6). Finally, each outside wall of the sheath and end of the opening of the pipe was washed with cell culture solution, and the solution was evaluated for cytologic evidence to determine the presence of muscle fragments before re-inserting it into the abdominal cavity. Cytological findings were obtained using an Auto Cycle Prep 2002 instrument (Becton, Dickinson and Company, Franklin Lakes, New Jersey, USA). Positive results were considered to have resulted from leakage during the laparoscopic procedure.

The outcomes were (i) leakage rates, (ii) bag introduction time (time from system insertion to morcellation start), (iii) bag removal time (time from the completion of specimen removal to the completion of bag removal), and (iv) in-bag morcellation time. Statistical analyses were performed using SPSS software (version 23) (IBM Company, 1 New Orchard Road Armonk, New York, United States). Data are presented as medians (range) for non-parametric continuous variables; non-parametric data were analyzed using Mann–Whitney U-tests at a significance level of *p* ≤ 0.05.

## Results

In the experimental group, we found a tear in one case when the gas had not been completely discharged during bag removal. In the control group, leakage was found in eight cases, five positive with the two methods. Seven leaks were in the soft pipe, and one was in the bag. Although the tear is tiny, when we use white gauze to wipe the bag's body and pipe, the tear can still be observed through the gauze blue dyeing. The blue stain on the inner wall of the laparoscopic training box and trocar can confirm the leakage. In the control group, muscle fragments were found in both the opening of the umbilical pipe and the inner wall of the trocar (5/30). In the experimental group, the cytological test was negative in the pipe opening and the trocar's outside wall (0/30). The leakage rate was 0.03% and 26.6% in the experimental and control groups, respectively (*p* < 0.05; Table 2 Leakage of the system). The morcellation time was significantly shorter for the experimental group than that for the control group (9 min vs 13.5 min, *p* < 0.001), and the median time of bag introduction (7 min vs 9 min, *p* < 0.001) was shorter in the experimental group than that in the control group. However, although the removal times tended to be shorter in the experimental group, the difference was not significant (2 min [1.5–2.5 min] vs 2 min [2–3 min], *p* = 0.293; Table 3 Contained tissue extraction).

**Table 2:** Leakage of the system

	Experimental group	Control group	*p*-value
Trainer box visual inspection	1/30 (Trial 1)	7/30 (Trial 4, 9, 13, 14, 18, 27, and 30)	
Bag visual inspection	0/30	2/30 (Trial 6 and 9)	
Cytological findings	0/30	5/30 (Trial 6, 9, 13, 18, and 27)	
Leakage rate	3.3%	26.6%	0.026

**Table 3:** Contained tissue extraction

	Experimental group	Control group	*p*-value
Bag introduction time^a^	7 min (range, 4–8.5 min)	9 min (range, 7.5–12 min)	< 0.001
In-bag morcellation time^a^	9 min (range, 8–10 min)	13.5 min (range, 9–15 min)	< 0.001
Bag removal time^a^	2 min (range, 1.5–2.5 min)	2 min (range, 2–3 min)	0.293

Bag introduction time (time from system insertion to morcellation start); bag removal time (time from the completion of specimen removal to the completion of bag removal)

^a^Media**n** time

## Discussion

Iatrogenic tumor dissemination and planting seriously endanger patients' health. However, this should not negate the value of laparoscopic technology and limit the development of surgery from majorly invasive to minimally invasive. Subsequently, various tissue containment systems have been developed.

First, the leakage rate is related to the bag material used in the system. Prasanna Hariharan et al. [11] have tested seven brands of tissue containment bags with varying materials to evaluate the ability of bags to remain impermeable to blood and cancer cell surrogates under surgery. They demonstrated that the performance of the composite bags appeared to be dependent on the thickness of the polymer layer of the composite mesh. Only one of the seven bags with the thinnest polymer layer (0.03 mm) leaked during the dye tests. In our study, the bag of the control group was made of the homogeneous polyurethane(0.05 mm) as those of the experimental group(0.06 mm), and the thickness was basically the same to minimize the difference in leakage rate caused by the difference in bag material (Table 1).

In 2014, Cohen et al. [12] demonstrated that for single-site and multi-port techniques, a 1-piece isolation bag successfully contains tissue during the morcellation process. They reported a single puncture site in 13 trials when using a stitch-sealed bag. In 2015, Rimbac et al. [13]showed the technical feasibility and safety of an in-bag morcellation system in a clinical setting during laparoscopic hysterectomy. Two of the six bags developed tiny leaks at the point of optical mirror insertion [13]. A subsequent prospective in vivo study reported spillage in seven of 76 cases (9.2%) [14]. In one case, a containment system tear was noted before morcellation owing to manipulation of the bag during insertion. In another case, a tear was created during containment system removal.

Based on these studies, we believe leakage mainly occurs during laparoscopic sheath re-insertion and bag removal. There might be several reasons for this: 1. After the soft pipe was removed from the abdominal wall, the channel was blocked due to the abdominal wall's contraction. When the laparoscopic sheath was re-inserted, the change in insertion angle caused damage. 2. The soft pipe was twisted easily, and the laparoscopic sheath was blocked when re-inserted, which could also cause damage. After the rigid pipe in the experimental group was removed from the abdominal wall, it will not be affected by the contraction of the abdominal wall and will remain unobstructed. The bag could also become twisted in the experimental group but can be discovered under the supervision and corrected by turning the rigid pipe, thereby avoiding damage. The integrity of morcellation bags can also be impaired after morcellation, even if the bags appear to be intact during the procedure [15, 16]. After removing the system, a tear was found in the pipe in seven cases in the control group. In the experimental group, only one tear was found with the removal of the bag when the bag was not completely deflated. In subsequent experiments, the system was not removed until the bag was completely deflated and there was no leakage.

Leakage also occurs when the umbilical pipe is closed by two knots and removed through the abdominal cavity. Because tissue may contaminate the pipe opening when the optical mirror is removed from the system, the knotting method only closes the middle part of the pipe and cannot prevent tissue contamination at the end of the pipe opening. When the pipe is re-inserted into the abdominal cavity, tissue debris may contaminate the abdominal cavity and the right channel. Here, we used three methods to monitor for leakage; any positive result was considered a leak. The pipe's opening was checked with cytological tests for the first time to evaluate the airtightness of the system. In the control group, the blue dye stained the inner wall of the trocar sleeve and the outside of the knotted pipe. The cytology test was positive, indicating that knotted closing methods cannot effectively prevent leakage. The new system has made an important improvement, and the rigid pipes were all equipped with a sealing cap, which prevents tissue discharge from the top of the pipe when taken back into the abdominal cavity. The only case of leakage in the experimental group occurred when the gas had not been completely discharged during bag removal. In all subsequent experiments, no leakage occurred when the gas was discharged entirely before the bag body was removed. Thus, the design of the sealing cap can effectively avoid leakage.

In a study by Devassy et al., the mean bag manipulation and morcellation times were 7 and 12 min, respectively [17]. In this study, the bag manipulation time of the experimental group was similar to that reported previously. However, the morcellation time was significantly shorter for the experimental group than that for the control group (9 min vs 13.5 min, *p* < 0.001). This is because the control group only had an optical pipe and a pipe for morcellation. The new system in the experimental group has an extra auxiliary pipe; thus, an assistant collaborated on the morcellation, and the morcellation speed was significantly improved. The median bag introduction (7 min vs 9 min, *p* < 0.001) and removal times were shorter in the experimental group than in the control group. However, although the removal time tended to be shorter in the experimental group, the difference was not significant (2 min (1.5–2.5 min) vs 2 min (2–3 min), *p* = 0.293). After the system was inserted into the abdominal cavity, the bag needed to be opened manually with forceps in the control group. It is easy to confuse the inside and outside of the bag, as the bag opening is unmarked. Thus, the tumor tissue cannot be placed into the bag correctly and quickly. In the experimental group, the bag's opening was joined with an elastic titanium alloy ring that can be easily opened and closed and has a blue logo for identification. The detachable rigid pipe can be easily connected with multiple trocar channels to form a new airtight device with the aid of the anti-slip and conformal fixing forceps, which is similar to a traditional laparoscope and compatible with the laparoscopic power morcellator proposed by the FDA.

The rigid pipe is equipped with a bullet-shaped sealing cap with a small ring on the top, which is convenient for pipe removal from the abdominal incision with forceps. The anti-slip device at the junction of the rigid pipe and bag can prevent the bag from slipping into the abdominal cavity when pulling the pipe. If it is used clinically, especially for giant tumors, obese patients, and people with limited abdominal cavity volumes, these advantages will be clear. We believe the rigid pipe is made of a hard material, making it easier to insert and remove the containment bag through the operating hole than a soft bag alone, thereby shortening the time required for bag manipulation and removal.

The limitations of this study include small sample size and the lack of in vivo experimental data and long-term outcome information. Consequently, the results of this study must be interpreted with caution, and further investigation is needed for a full assessment of the new system.

## Conclusion

In 2020, the FDA recommended performing laparoscopic power morcellation with a laparoscopic power morcellation containment system when morcellation is appropriate [6]. However, different containment systems still have different degrees of tissue leakage in clinical use. In our in vitro study, the system with multiple detailed designs exhibited a lower leakage rate and enhanced the convenience of operation. These results provide a better way to achieve the problem of removing large tissues in minimally invasive surgery under the tumor-free principle. However, prospective clinical studies with larger cohorts are necessary to confirm the safety and convenience of the technology during laparoscopic morcellation.

## Data Availability

All data generated or analysed during this study are included in this published article [and its supplementary information files].

## Abbreviations

FDA: Food and Drug Administration
Co. LTD: Company Limited
USA: United States of America
SPSS: Statistical Product and Service Solutions
IBM: International Business Machines Corporation
